# Targeting HMGB1-NFκb Axis and miR-21 by Glycyrrhizin: Role in Amelioration of Corneal Injury in a Mouse Model of Alkali Burn

**DOI:** 10.3389/fphar.2022.841267

**Published:** 2022-05-02

**Authors:** Peihong Wang, Peng Hao, Xi Chen, Linghan Li, Yongying Zhou, Xiaohan Zhang, Lin Zhu, Ming Ying, Ruifang Han, Liming Wang, Xuan Li

**Affiliations:** ^1^ Clinical College of Ophthalmology, Tianjin Medical University, Tianjin, China; ^2^ Tianjin Eye Hospital, Tianjin Key Laboratory of Ophthalmology and Visual Science, Tianjin Eye Institute, Tianjin, China; ^3^ Nankai University Affiliated Eye Hospital, Tianjin, China

**Keywords:** HMGB1, glycyrrhizin, corneal inflammation, neovascularization, MicroRNA-21, corneal opacity

## Abstract

Corneal neovascularization (CNV) is a sight-threatening condition usually associated with various inflammatory settings including chemical injury. High mobility group box 1 (HMGB1) is identified as an inflammatory alarmin in diverse tissue damage. Here, we evaluate the expression of HMGB1 and the consequences of its inhibition through its selective inhibitor glycyrrhizin (GLY) in alkali burn-induced corneal inflammation and neovascularization. GLY effectively attenuated alkali burn-induced HMGB1 expression at both mRNA and protein levels. Furthermore, slit-lamp analysis, ink perfusion, H&E staining, and CD31 histochemical staining showed that GLY relieved corneal neovascularization, while GLY attenuated VEGF expression via inhibiting HMGB1/NF-κB/HIF-1α signal pathway. In addition, GLY treatment decreased the cytokine expression of CCL2 and CXCL5, accompanied by the reduction of their receptors of CCR2 and CXCR2. GLY diminished the inflammatory cell infiltration of the cornea, as well as reduced the expression of IL-1β, IL-6, and TNF-α. Moreover, treatment with GLY reduced the degree of cornea opacity through inactivating extracellular HMGB1 function, which otherwise induces TGF-β1 release and myofibroblast differentiation. Furthermore, we found that GLY treatment attenuated the upregulation of miR-21 levels in alkali burned cornea; while inhibition of miR-21in keratocytes *in vitro*, significantly inhibited TGF-β1-induced myofibroblast differentiation. Collectively, our results suggested that targeting HMGB1-NFκb axis and miR-21 by GLY could introduce a therapeutic approach to counter CNV.

## Introduction

The cornea is the outmost tissue of the eye, which is transparent and avascular to maintain the clarity of vision. When blood vessels invade into the normally avascular cornea, namely corneal neovascularization (CNV), could result in tissue scarring, lipid accumulation and corneal edema (Chang et al., 2001). CNV can result from various etiologies including the following four groups: hypoxia (contact lens wear), corneal infections, ocular surface inflammation and injury including limbal stem cell deficiency (LSCD). Under normal conditions, corneal avascularity is retained by the balance between angiogenic and antiangiogenic factors. Once this balance turns to angiogenic factors, CNV appears (Cursiefen et al., 1998). Vascular endothelial growth factor (VEGF) has been supposed to be one of the major angiogenic factors, thus anti-VEGF therapy happens to be an effective treatment strategy by directly inhibiting angiogenesis at the molecular level (Ozdemir et al., 2013). However, the virtue of anti-VEGF therapy is limited and varies among patients (Keating and Jacobs, 2011). Mirabelli et al. found that topically application of anti-VEGF drugs decreases the growth of corneal vessels by only 14% in an inflammatory model in rat, possibly due to targeting VEGF-A does not directly interfere with the associated inflammation (Mirabelli et al., 2014). Nevertheless, inflammation is the center of CNV induced by various etiology including chemical injury. Alternative ways able to block both the inflammation and angiogenesis are therefore of therapeutic interest.

Corneal alkali burns are characterized by injury-induced inflammation, neovascularization, and fibrosis, represent one of the most devastating injuries to the eye. Cornea alkali burns belong to one of the acquired causes of CNV. Crosstalk between inflammatory cells (such as macrophages and neutrophils) and angiogenic growth factors (VEGF family) in inflammation induced CNV is apparent. Following corneal alkali burns, inflammatory cells such as leukocytes, macrophages, activated keratocytes, and mesenchymal cells such as myofibroblasts, and neovascularization factors are activated. Early treatment with anti-inflammatory therapy is critical in preserving eye integrity, decreasing CNV and improving corneal clarity.

High mobility group box 1 (HMGB1) has been identified as a nuclear non-histone DNA-binding protein, which functions to stabilize nucleosome structure as well as to regulate transcription (Li et al., 2003; Ulloa and Messmer, 2006). It can be passively released or actively secreted into the extracellular milieu form dead cells and inflammatory cells. HMGB1 serves as typical damage-associated molecular pattern (DAMP), participating in various systemic inflammations such as sepsis, arthritis and autoimmune diseases (Wang et al., 1999; Scaffidi et al., 2002). It functions by binding to cellular receptors such as the receptor for advanced glycation end products (RAGE), toll-like receptor (TLR)-2 and TLR-4 (van Zoelen et al., 2009). It was reported that HMGB1 can regulate the activation of endothelial cells through interacting with RAGE, which eventually leads to NF-κB activation. Reports showed the role of NF-κB in angiogenesis through regulating VEGF expression and the inflammatory response (Abeyama et al., 2000; Luan et al., 2010). Activated NF-κB accelerates transcription of target genes, including TNF-α, chemokine (C–C motif) ligand 2 (CCL2; MCP-1) and C-X-C motif chemokine 5 (CXCL5; ENA78)(Kaulmann and Bohn, 2014). These factors can induce monocyte and neutrophil infiltration into inflammation sites and could further binds to their cell surface receptors such as CXCR2 for CXCL5 and CCR2 for CCL2 to activate NF-κB signaling in turn. Furthermore, HMGB1 could induce both leukocytes and immune cells to release cytokines like IL-1β (Emilie, 2012; Zhou et al., 2020), which is a significant cytokine in inflammatory relevant angiogenesis (Nakao et al., 2005) and also enhances production of strong pro-angiogenic molecules VEGF and bFGF (Salven et al., 2002).

Glycyrrhizin (GLY), one of the major components of G. glabra, has been reported to possess various of pharmacologic functions such as anti-inflammatory, antiangiogenic, anti-viral, anticancer, and hepatoprotective activities (Tan et al., 2018). GLY inhibits angiogenesis and tumor growth *via* inhibiting cell motility and immunological response (Smolarczyk et al., 2012). Furthermore, GLY binds to HMGB1 and counteracts its chemokine and cytokine mediated inflammatory cascade (Wu et al., 2015). It was reported that GLY treatment effectively reduced HMGB1 expression at both mRNA and protein level in infection or non-infection corneal injury and thus to improve corneal repair (Ekanayaka et al., 2016; Zhou et al., 2020).

Emerging evidence suggests that microRNAs (miRNAs) play important roles in diverse physiologic and pathologic processes, including development, differentiation, proliferation, apoptosis, and carcinogenesis. Chen et al. has reported that HMGB1 promotes hepatocellular carcinoma progression through miR-21-mediated MMP activity (Chen et al., 2015). MiR-21 has been associated with wound healing in corneal epithelial cells (Zhang et al., 2019). However, the effect of GLY on miR-21 in corneal wound healing is not clear.

Therefore, this study aimed to investigate the potential role of HMGB1 in the aberrant inflammation relevant to corneal neovascularization and opacity. Consequently, we have investigated *in vivo* whether application of the selective inhibitor GLY to inactivate HMGB1 activity could affect the outcome of inflammatory CNV. Finally, the mechanism of GLY in inflammatory corneal neovascularization and the effect of GLY on HMGB1-NFκb axis and miR-21 were investigated.

## Materials and Methods

### Mice

Male C57BL/6J mice aged 8–10-weeks were purchased from the Institute of Haematology and Blood Diseases Hospital, Chinese Academy of Medical Sciences & Peking Union Medical College (Tianjin, China). The mice were checked under a slit-lamp, and those with anterior segmental lesions were excluded. Animals were maintained in a standard condition where a 12 h light/dark cycle at 22–24°C with 45–50% humidity is available. All experiments procedures involving animals were performed in accordance with the Association for Research in Vision and Ophthalmology (ARVO) Statement for the Use of Animals in Ophthalmic and Vision Research. The animal study was reviewed and approved by the Animal Care and Use Committee of Tianjin Eye Hospital.

### Alkali-Burn-Induced Inflammatory Corneal Neovascularization

Mice were anaesthetized intraperitoneally with pentobarbital sodium (70 mg/kg) followed by topical anesthesia of proparacaine hydrochloride (Santen, Suzhou, China), then excess liquid was wiped off with a cotton swab and a 2 mm diameter filter paper (dipped in 1 mol/L NaOH for 20s) was placed on the center of the right cornea for 40s under a surgical microscope. Next, the paper was removed immediately and the eye surface was gently rinsed with normal saline (NS) for 2 min and levofloxacin eye drops were used to prevent infection. All the alkali burn model was grad I of Dua et al.‘s classification (Dua et al., 2001). The left eyes were used as normal controls.

### Glycyrrhizin or PBS Treatment

After establishing the alkali-burn mice model, the mice were randomly distributed into two groups (GLY- or PBS-group) and the right eyes of each mouse were topically instilled with 5 μL 2 μg/μL GLY (Sigma-Aldrich Corp, United States) or PBS every 4–6 h for 14 consecutive days and then twice a day for another 14 days. At 0, 2, 4, 7, 14, 28 d, slit-lamp images were managed and the growth of corneal neovascularization was measured as the length of the longest and the most straight new vessels by the vernier caliper. The area of new vessels was analyzed by the formula: CNV(mm^2^) = C/12 × *π* × [r^2^-(r-l)^2^], C is the number of corneal peripheral hours involved by CNV, r = 1.5 mm (the radius of the mice cornea), l is the length of new vessels.

### Biomicroscopic Grading of Corneal Opacity

Corneal opacity was observed at 28 days after alkali-burn and graded on a numerical scale of 0–4: 0, completely clear cornea; 1, mild stromal opacity with iris clearly visible; 2, moderate stromal opacity with iris vaguely visible; 3, severe corneal opacity with looming visible iris; 4, opaque cornea with iris not visible (Yoon et al., 2008). Three ophthalmologists observed the corneal opacity in a blinded manner separately.

### Ink Infusion

Mice were deeply anaesthetized intraperitoneally with pentobarbital sodium (70 mg/kg) and the heart was completely exposed. Then the perfusion needle is injected into the left ventricle with 20 ml normal saline, while the right atrium is cut open to drain out the blood and perfusion fluid. Next, a mixture of gelatin-Indian ink-normal saline synthetic color gel (20 ml) was followed to perfuse new blood vessels (Wang et al., 2016). The head was immersed into ice for 5 min and then the eyeballs were corneas were separated. The collected corneas were fixed in 4% paraformaldehyde for 2 h, gradient alcohol dehydration (80% overnight, 95% 2 h for 2 times, anhydrous ethanol for 2 times), cut four knives with a blade under the microscope, smooth the corneas, seal the slides, and take pictures. The images were analyzed by Image-Pro Plus 6.0.

### Histological, Immunofluorescence and Immunohistochemical Staining

Eyes were collected at 0, 2, 4, 7, 14, 28 days after alkali-burn from GLY- or PBS-applied as well as untreated normal control mice. The eyes were then kept in paraformaldehyde solution (40 g/L) for at least 24 h at room temperature, gradient alcohol, xylene and embedded in paraffin for the final sectioning with the thickness of 4 μm. Tissue sections stained with haematoxylin/eosin (HE) were used to examine the corneal structure as well as infiltration of neutrophils and neovascularization. In addition, tissue sections were stained using immunofluorescence staining for NF-κB-p65 and immunohistochemical staining for HMGB1, CD31, myeloperoxidase (MPO), TGF-β1, α-SMA, fibronectin, collagen III. Briefly, sodium citrate repair solution by microwave heating was performed for antigen retrieval, 3% H2O2 was employed for non-specific antigens elimination, and then the sections were immersed in normal goat serum for 2 h for blocking and subsequently incubated with primary antibodies specific for NF-κB-p65 (1:1000 dilution, no11014, SAB Signalway Antibody), HMGB1 (1:1000 dilution, ab18256, Abcam), CD31 (1:1000 dilution, ab245689, Abcam), MPO (Ready-to-use, GT203207, Gene Tech), TGF-β1 (1:1000 dilution, sc-52893, Santa Cruz Biotechnology), fibronectin (1:1000 dilution, sc-9068, Santa Cruz Biotechnology), α-SMA (1:2000 dilution, no55135-1-AP, Proteintech Group) and collagen III (1:1000 dilution, sc-271249, Santa Cruz Biotechnology) overnight at 4°C. The next day, the sections were incubated with the Envision horseradish peroxidase system (Gene Tech, Shanghai, China) or fluorescent secondary antibody (1:5000 dilution) for 2 h under room temperature. The sections were finally incubated for 5 min with 3,3′-diaminobenzidine (DAB) (Gene Tech, Shanghai, China) or 4',6-diamidino-2-phenylindole (DAPI 1:1000, Sigma). Protein expression was observed and photographed with a microscope. Neutrophils were counted as follows: corneal sections were randomly selected from each group, and the number of neutrophils positive with MPO staining in four non-repeated fields was randomly counted under a 200-fold microscope, and then the average was the number of neutrophils.

### Cell Culture and Treatment

Human keratocytes (HK, catalog #6502) and culture medium (Fibroblast Medium, FM, Catalog #2301) were purchased from sciencell (sciencell, Carlsbad, CA, United States). HK were cultured in the culture medium according to the manufacture’s instruction. Briefly, HK from passages 4-7 were subjected to serum starvation for 24 h before being used for the experiments. HK were treated with TNFα (10 ng/ml) and TGF-β1 (5 ng/ml) to induce inflammation or fibrosis, respectively. To evaluate the effects of GLY, HK treated with or without TNFα was incubated with 0.1 mM GLY for 24 h. For the transfection experiment, the miR-21-5p agomir, MiR-21-5p antagomir and their respective negative controls (NC) were obtained from GenePharma (Shanghai, China). Transfection of HKs was performed using Lipofectamine 3000 Transfection Reagent (Invitrogen, Carlsbad, CA) according to the manufacturer’s instructions. The agomir of miR-21-5p used for overexpression was double stranded with the following sequence: Forward, 5'-UAG​CUU​AUC​AGA​CUG​AUG​UUG​A-3'; Reverse, 5'-AAC​AUC​AGU​CUG​AUA​AGC​UAU​U-3'. The miR-21-5p antagomir was single stranded with the following sequence: 5'-UCA​ACA​UCA​GUC​UGA​UAA​GCU​A-3'. HK were cultured in six-well plates at 70% confluence at the time of transfection. Sixteen hour after transfection, the culture medium was replaced by fresh medium with TGF-β1.

### RT-qPCR

Total RNA of the corneas was extracted (*n* = 4) with TRIzol (Invitrogen, Carlsbad, CA, United States) following the manufacturer’s protocol. Total RNA amount was quantified by the machine (NanoDrop One, Thermo Fisher Scientifc) and reverse transcripted to cDNA following the manufacturer’s protocol (M-MLV reverse transcriptase kit (Promega Corporation, Madison, WI, United States). RT-qPCR was performed using SYBR Premix Ex Taq™ (Takara Biotechnology Co., Ltd., Dalian, China) and the LightCycler®96 SW1.1 PCR system (Roche, Germany). Relative expression values of target genes were normalized to β-actin, and fold change was computed using the relative quantification (2-ΔΔCT) method. Three technical replicates in each group were performed. The primer sequences were as follows: VEGF (forward: AAA​ACA​CAG​ACT​CGC​GTT​GC, reverse: GGT​CTT​TCC​GGT​GAG​AGG​TC), TNF-α (forward: CGA​GTG​ACA​AGC​CTG​TAG​CC, reverse: TTG​AGA​TCC​ATG​CCG​TTG​GC), CCL2 (forward: CCA​ATG​AGT​AGG​CTG​GAG​AGC, reverse: ACC​CAT​TCC​TTC​TTG​GGG​TC), CXCL2 (forward: AAG​GCA​AGG​CTA​ACT​GAC​CTG, reverse: TTG​GTT​CTT​CCG​TTG​AGG​GAC); IL-1β (forward: TGC​CAC​CTT​TTG​ACA​GTG​ATG, reverse: TGA​TGT​GCT​GCT​GCG​AGA​TT), IL-6 (forward: TTT​CTG​GGC​AAA​CTG​TTA​CCG, reverse: GTG​GGG​AAG​TGG​CAA​CTG​AT), HIF-1α (forward: TTT​CTG​GGC​AAA​CTG​TTA​CCG, reverse: GTG​GGG​AAG​TGG​CAA​CTG​AT), CXCR2 (forward: CAG​CTG​GTG​CCT​CAG​ATC​AA, reverse: ATC​TCC​AGT​GGG​CAG​CAT​TC); CCR2 (forward: AGG​AGC​CAT​ACC​TGT​AAA​TGC​C, reverse: TGT​CTT​CCA​TTT​CCT​TTG​ATT​TGT) and beta-Actin (forward: GAT​TAC​TGC​TCT​GGC​TCC​TAG​C, reverse: GAC​TCA​TCG​TAC​TCC​TGC​TTG​C).

### Western Blot Analysis

Corneas (*n* = 4) were harvested at 2, 4, 7, 14, 28 days and ground and then homogenized in RIPA lysis buffer containing a mixture of protease and phosphatase inhibitor. Lysates were then centrifuged at 12,000 × g for 10 min at 4°C and supernatants were harvested, and the concentration was valued with a bicinchoninic acid assay kit. Subsequently, samples were mixed with 5X SDS-PAGE loading buffer and boiled for 10 min at 100°C. Equivalent amount of protein were run in a 10–12% SDS-PAGE gel and then transferred to PVDF membrane. Membranes were cut properly and immersed in 5% milk for 2 h at room temperature, and then incubated overnight at 4°C with primary antibodies for HMGB1 (1:500 dilution, ab18256, Abcam), VEGF (1:1000 dilution, ABS82, Sigma Aldrich), TNF-α(1:1000 dilution, ab6671, Abcam), Fibronectin (1:1000 dilution, sc-9068, Santa Cruz Biotechnology), GAPDH (1:1000 dilution, ab181602, Abcam) and β-actin (1:1000 dilution, no4967, Cell Signalling Technology). On the following day, secondary antibody (1:5000 dilution) incubation was managed for 2 h at room temperature. Protein expression was measured by the Odyssey® Sa Two-colour infrared laser imaging system (Licor, United States). The images were analyzed by Image-Pro Plus 6.0.

### Statistical Analysis

All quantitative data were expressed as mean ± SD. The data were tested for normality with the Shapiro Wilk test or the Kolmogorov-Smirnov test. A Student t-test was used to assess the differences between the two means. The assessment of multiple means was performed by one-way or two way ANOVA followed by the Bonferroni’s post hoc test. In case of a low sample size (low n number) that was not sufficient for normality testing, nonparametric tests (the Mann- Whitney U-testor Kruskal-Wallis test) were used. All statistical analyses were performed using GraphPad Prism (version 8). Statistical significance was defined at *p* < 0.05.

## Results

### In Alkali Burn-Induced Inflammatory Corneal Neovascularization, HMGB1 Expression Is Upregulated, Whereas Glycyrrhizin Effectively Declines HMGB1 Release

**FIGURE 1:**
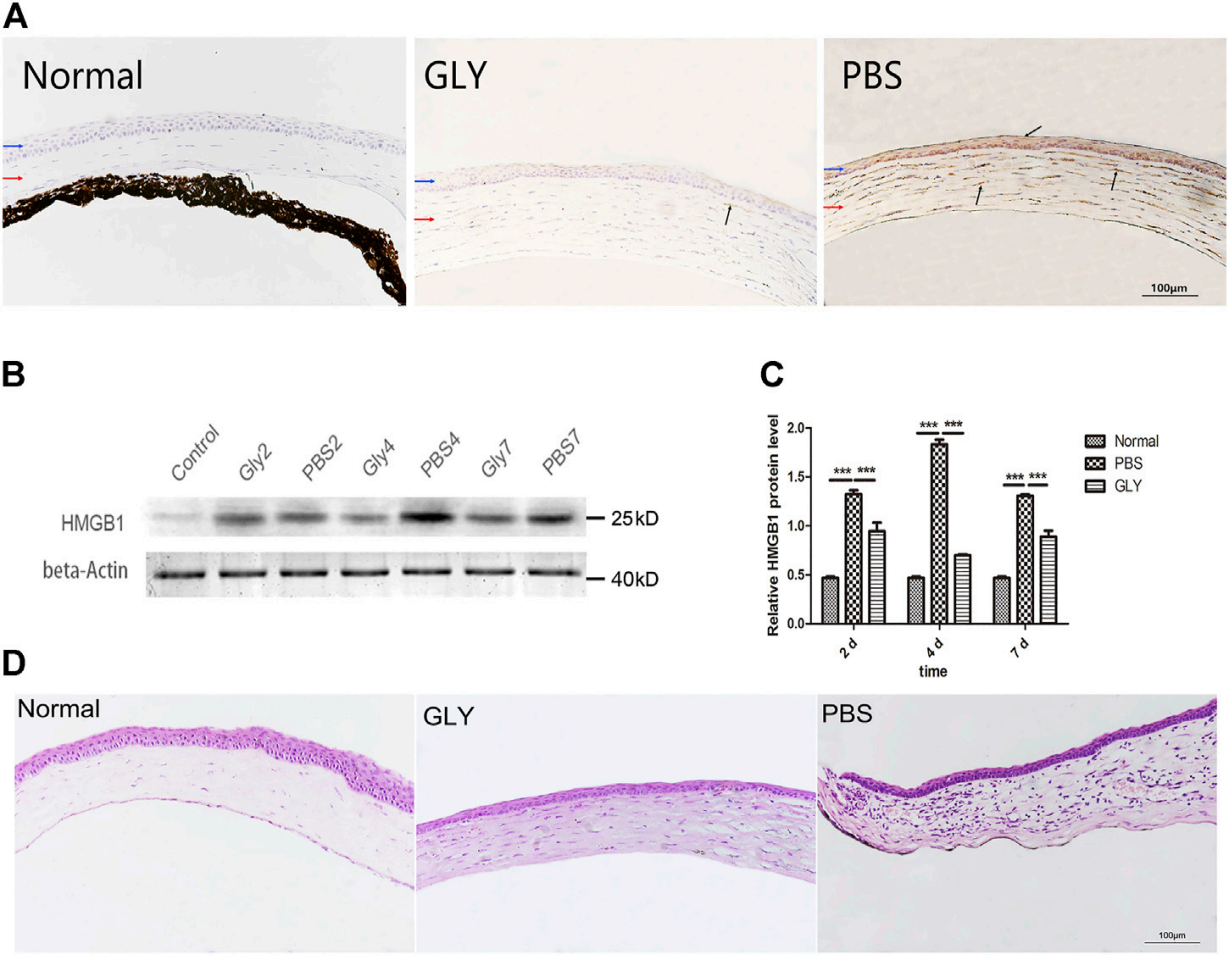
Expression of HMGB1 and the effect of GLY at different time points following corneal alkali burn. **(A)** Immunohistochemical staining of HMGB1 at day 7 after alkali burn. **(B)** Western blotting showed that the expression of HMGB1 was markedly reduced on days 2, 4, and 7 in the GLY-group compared with that of the PBS-group, and the quantified HMGB1 protein is shown in **(C)**. Data are shown as the mean ± standard deviation (SD) and are representative of three independent experiments. Data were analyzed using one-way ANOVA followed by Bonferroni’s multiple comparison test (*n* = 3/group). **(D)** Haematoxylin and eosin **(H,B)** staining showed the histologic appearance of the cornea at 14 d after alkali burn. Corneal parts are marked by arrows: blue arrow, corneal epithelium; red arrow, corneal stroma. Dark arrows show extranuclear or extracellular HMGB1 staining. Scale bar = 100 μm *p < 0.05, **p < 0.01, ***p < 0.001.

To determine whether HMGB1 is an effective modulator of the corneal neovascularization involved in the inflammation cascade, the present study first examined the expression of HMGB1 in the process of corneal neovascularization. Thus, we established an alkali burn-induced corneal neovascularization model on the right eyes of C57BL/6J mice. Cornea alkali burn caused a significant increase the expression of HMGB1 by immunohistochemistry staining (Figure 1A) and western blot assay (Figures 1B,C). Compared with the PBS-treated group, GLY effectively decreased HMGB1 levels (Figure 1A), and this was supported further by western blot analysis, which showed that GLY treatment attenuated alkali burn-induced HMGB1 expression at 2, 4, 7 days after corneal injury (Figures 1B,C). Moreover, HE staining showed that GLY reduced the infiltration of neutrophils and neovascularization in comparison with that of PBS application (Figure 1D). These results suggest that HMGB1 might be a potent modulator that participates in healing events during inflammatory corneal neovascularization. Moreover, in the present study, GLY was an effective inhibitor that attenuates HMGB1 release.

### Glycyrrhizin Declines the Corneal Neovascularization and Cornea Opacity

**FIGURE 2:**
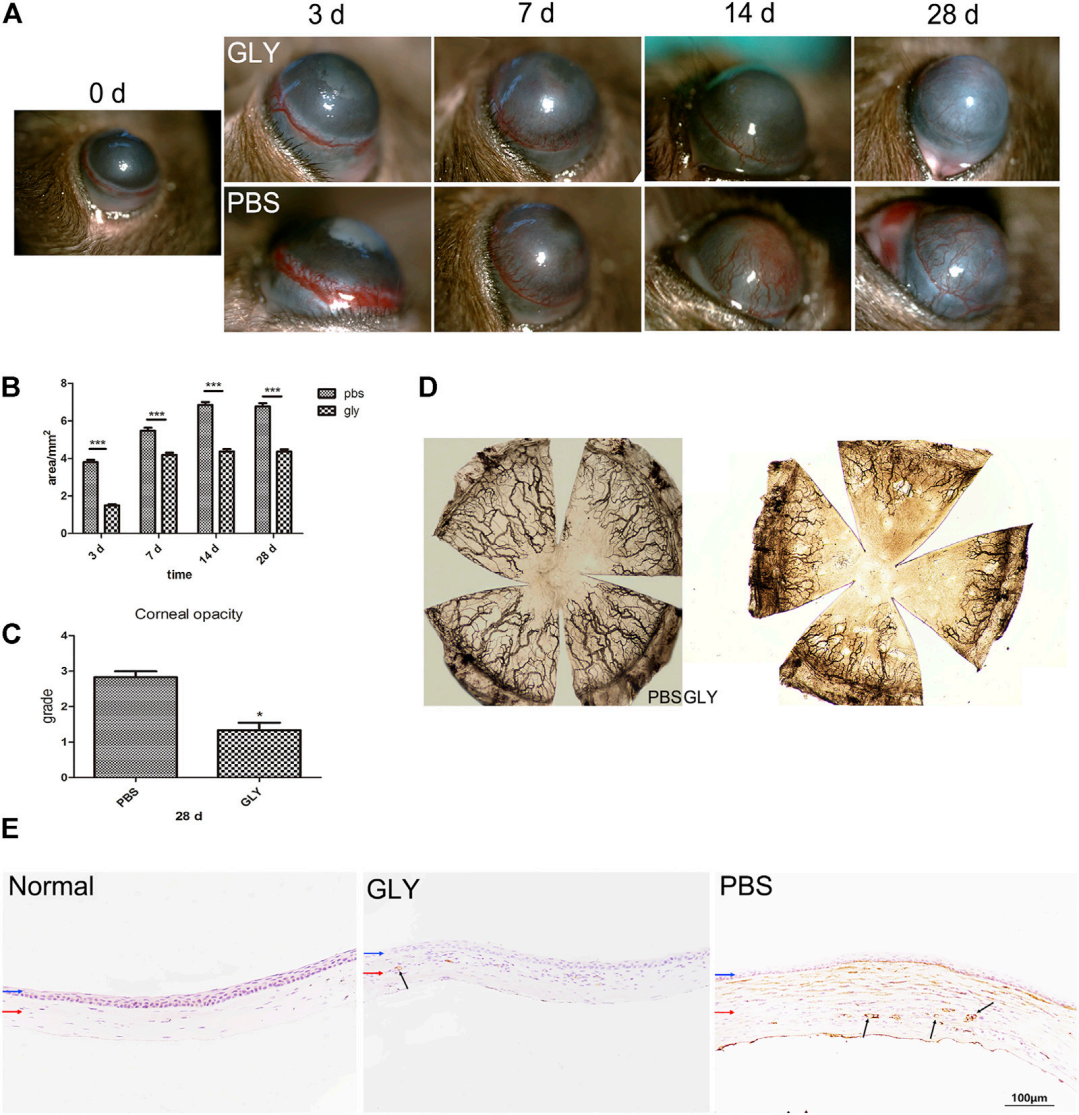
GLY inhibits corneal neovascularization and reduces haze opacity. **(A)** The corneal neovascularization was examined at 0, 3, 7, 14 and 28 d after the corneal alkali burn, and the quantified corneal neovascularization is shown in **(B)**. Data were analyzed using one-way ANOVA followed by Bonferroni’s multiple comparison test (*n* = 15/group). **(C)** Changes in corneal opacity over 28 d between groups. Data were analyzed using independent *t* test (*n* = 6/group). **(D)** The ink staining of corneas at 14 d. **(E)** Immunohistochemical staining for CD31 at 14 d after corneal alkali burn. Data are shown as the mean ± SD and are representative of three independent experiments. Blue and red arrows show corneal epithelium and cornea stroma, respectively. Dark arrows show the positive staining of CD31 cells. Scale bar = 100 μm *p < 0.05, **p < 0.01, ***p < 0.001.

To investigate the role of HMGB1 and GLY in inflammatory corneal neovascularization, we built the murine model of alkali-induce CNV followed by treatment with GLY or PBS. GLY, an effective inhibitor of HMGB1, was applied to inhibit HMGB1 functions. According to the slit-lamp images, CNV was restrained in GLY-treated mice when compared with that of PBS-treated mice (Figure 2A). By quantity analysis of the new vessels invaded areas of cornea, we found that the new vessels invaded areas of cornea in GLY-treated mice significantly less than those of PBS-treated mice on the day 3, 7, 14, and 28, respectively (Figure 2B). Moreover, ink staining provided further evidence that the percentage of vessels invaded areas of cornea in GLY-group mice was significantly decreased compared to that of PBS-group mice (Figure 2D). Furthermore, immunohistochemical staining for CD31, a marker for blood vessel endothelial cells, revealed similar tendencies in GLY group and PBS group (Figure 2E). These observations indicate that HMGB1 is a critical factor involved in alkali-induced CNV. In addition, cornea opacity at 28 days in the corneas of GLY group was markedly decreased by using GLY compared with that of PBS-group by haze grade analysis (Figure 2C). In general, we found that application of GLY inhibited corneal neovascularization, followed by decreased cornea opacity.

### HMGB1 Is a Significant Element That Modulates VEGF Expression in Inflammation-Mediated Corneal Neovascularization

**FIGURE 3:**
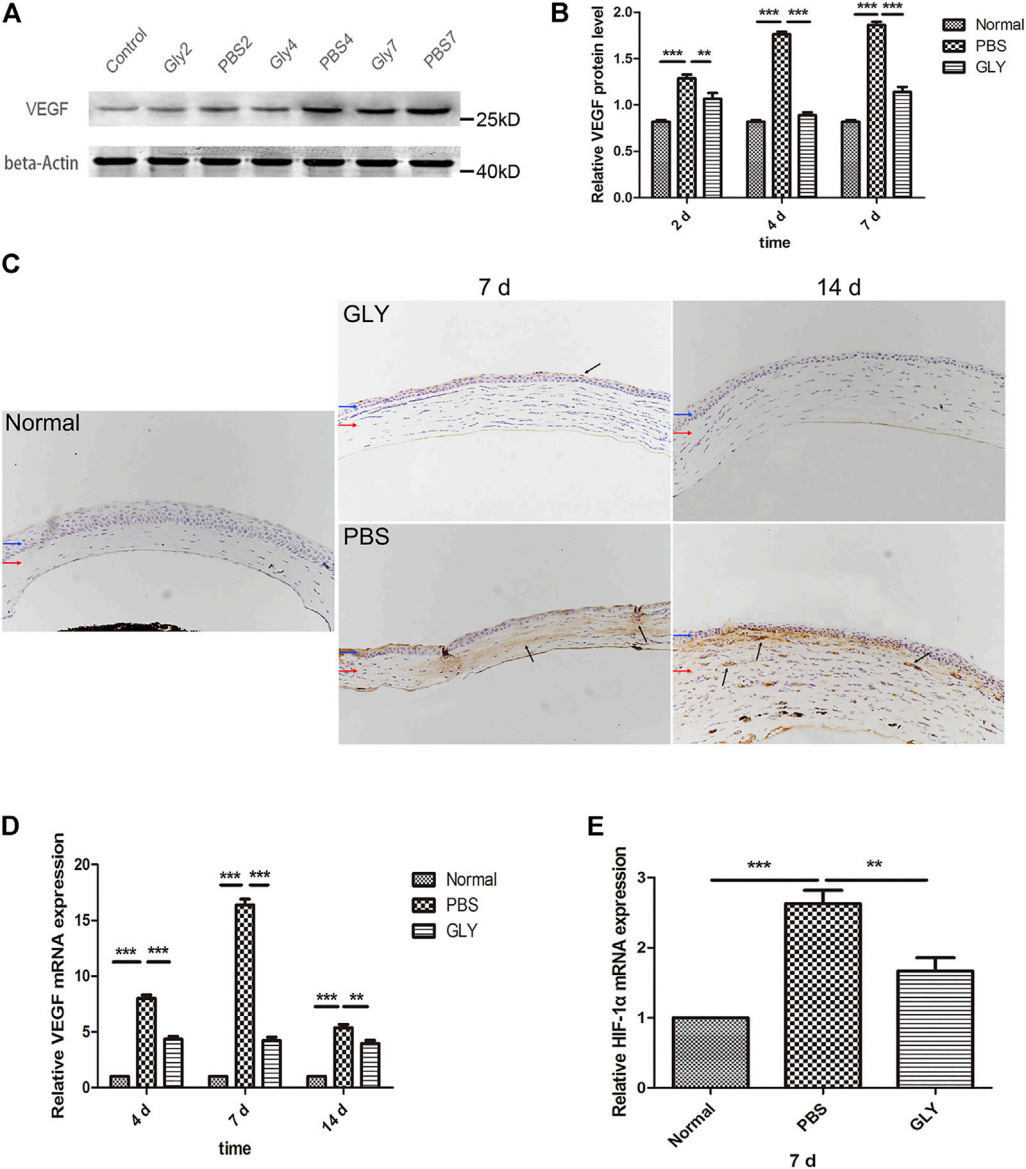
HMGB1 activates HIF-1α and VEGF expression. **(A)** Western blotting showed that extracellular HMGB1 activated VEGF expression in the groups, and the quantified VEGF protein is shown in **(B)** The results are shown as the mean ± SD and are representative of three independent experiments. Data were analyzed using one-way ANOVA followed by Bonferroni’s multiple comparison test (*n* = 3/group). **(C)** Immunohistochemical staining for VEGF (indicated by dark arrows) on day 7 and 14 after the corneal alkali burn. Scale bar = 100 μm. Blue and red arrows show corneal epithelium and cornea stroma, respectively. **(D)** mRNA expression levels of VEGF at different time points after corneal alkali burn. **(E)** mRNA expression levels of HIF-1α at 7 days after corneal alkali burn. Data were analyzed using one-way ANOVA followed by Bonferroni’s multiple comparison test (*n* = 6/group).The results are expressed as mean ± SD from three independent experiments and each experiment was performed in triplicate. *p < 0.05, **p < 0.01, ***p < 0.001.

**FIGURE 4:**
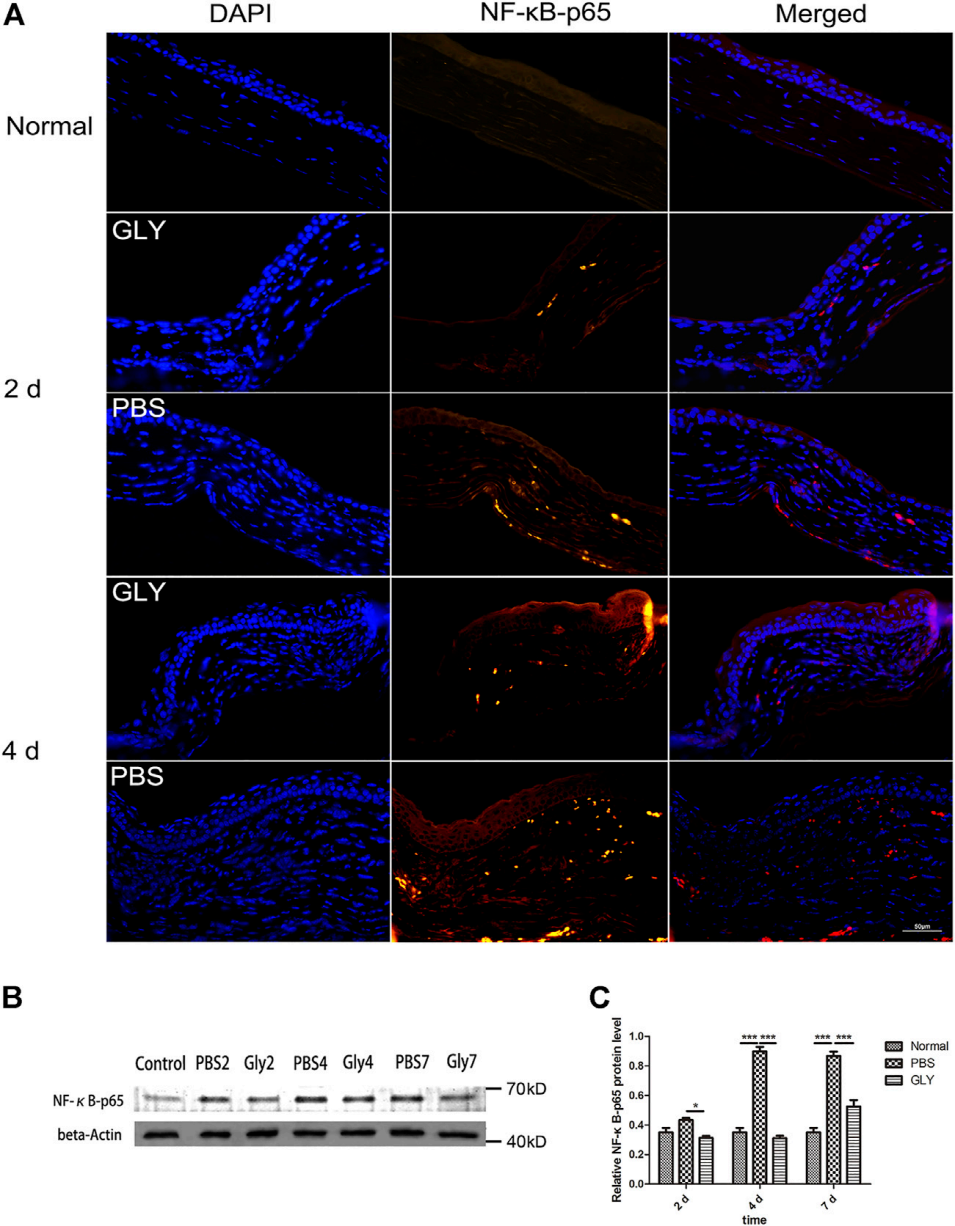
Extracellular HMGB1 activates NF-κB signaling pathway. **(A)** Representative positive NF-κB-p65 staining in the cornea form different groups by immunofluorescence on day 2 and day 4. **(B)** Western blotting showed that the expression of NF-κB-p65 was markedly reduced on days 2, 4, and 7 in the GLY-group compared with that of the PBS-group and the quantified NF-κB-p65 protein is shown in **(C)** Data were analyzed using one-way ANOVA followed by Bonferroni’s multiple comparison test (*n* = 3/group). The results are shown as the mean ± SD and are representative of three independent experiments. Scale bar = 50 μm *p < 0.05, **p < 0.01, ***p < 0.001.

Since the cornea is an avascular tissue in normal conditions, inflammation-induced hypoxia is more evident in corneal tissue (Stasinopoulos et al., 2009). It was proved that the inflammatory factors independent of hypoxia might be significant for HIF-1α activation in a mouse model of chronic contact lens wear (Chen et al., 2012). HIF-1α is a promoter of VEGF gene through binding to the hypoxia-responsive element (HRE), in which way up-regulating VEGF-A expression (Kimura et al., 2000). Furthermore, NF-κB is one of downstream targets of HIF-1α (Walmsley et al., 2005). The positive feedback promotes the inflammatory and angiogenic response. Consequently, as shown in Figure 3A, VEGF was obviously increased in the alkali-burned corneas compared with that in the healthy controls. This shift was rescued by GLY treatment (on days 2, 4, and 7). Quantitative analysis of the western blots also confirmed this (Figure 3B). Moreover, immunohistochemical analysis revealed that GLY reduced alkali burned-induced the expression of VEGF (Figure 3C). Further RT-qPCR analysis of VEGF also confirmed this (Figure 3D). In addition, we detected the expression of NF-κB-P65 and HIF-1α separately. The results of RT-qPCR assay showed that HIF-1α expression in the PBS group was obviously increased on day 7 compared with that in the healthy controls, whereas this change was reversed by GLY treatment (Figure 3E). In addition, immunofluorescence analysis showed that NF-κB-P65 was obviously transferred from the cytoplasm to the nucleus in the PBS-treated group in comparison with that in the healthy controls, while this transposition was rescued by GLY treatment (on days 2 and 4) (Figure 4A). Furthermore, western blot detection also showed that GLY treatment attenuated alkali-burn-induced of NF-κB-P65 translocation to nucleus in corneas (Figures 4B,C). Above all, the data imply that HMGB1 regulates the expression of VEGF at the protein and mRNA levels through NF-κB signaling. Furthermore, GLY was an effective regulator to inhibit VEGF expression.

### HMGB1 Calls Neutrophil Influx Into the Corneal Stroma to Exacerbate Inflammation and Angiogenesis

**FIGURE 5:**
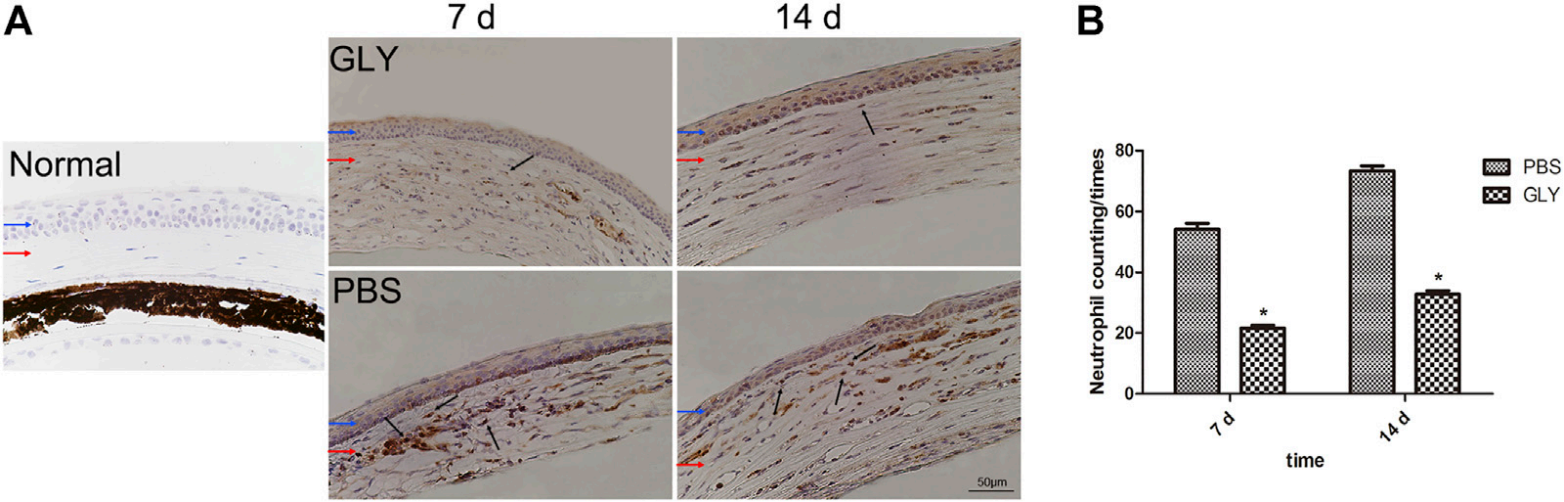
HMGB1 mediates chemokine-mediated neutrophil migration and infiltration. Neutrophil migration and infiltration levels were examined with immunohistochemical staining of MPO at 7 d and 14 d after the corneal burn in the groups **(A)**. MPO-positive cell number was significantly lower in GLY-group than PBS-group **(B)**. The results are shown as the mean ± SD and are representative of three independent experiments. Data were analyzed using one-way ANOVA followed by Bonferroni’s multiple comparison test (*n* = 6/group). Blue and red arrows show corneal epithelium and cornea stroma, respectively. Dark arrows show MPO-positive cells. Scale bar = 50 μm *p < 0.05.

After alkali-burn, there was a flow of inflammatory cells into the corneal stromal at early stage (Wilson et al., 2001), where neutrophils comes as the first significant population of leukocytes. Neutrophils have been shown to have dual functions as they not only functions as scavenger for cellular waste released from dead cells but also exacerbate uncontrolled or chronic inflammation (Kolaczkowska and Kubes, 2013). In addition, HMGB1 triggers the infiltration of neutrophils and induces a neutrophil-mediated injury amplification loop (Huebener et al., 2015). We studied the DAMP role of HMGB1 and its function in neutrophil-mediated inflammation in alkali-burn induced corneal inflammation and neovascularization *in vivo*. We confirmed that GLY treatment significantly decreased the protein or mRNA level of HMGB1 and VEGF, and thus, we mean to further study the effects on chemotactic cytokines and neutrophilic infiltration. Firstly, we measured myeloperoxidase, a neutrophil specific protein, to measure the amount of neutrophils in the corneal stroma. Immunohistochemical analysis showed there was a large quantity of neutrophil infiltration in the corneal stroma of PBS-group in comparison with that in the unwounded corneas, while GLY treatment rescued the level of neutrophil infiltration. Further neutrophil counting also confirmed the same on the day 7 and 14 (Figures 5A,B).

### HMGB1 Mediates the Expression of the Inflammatory Cytokines and Chemokines in Corneal Wound Healing

**FIGURE 6:**
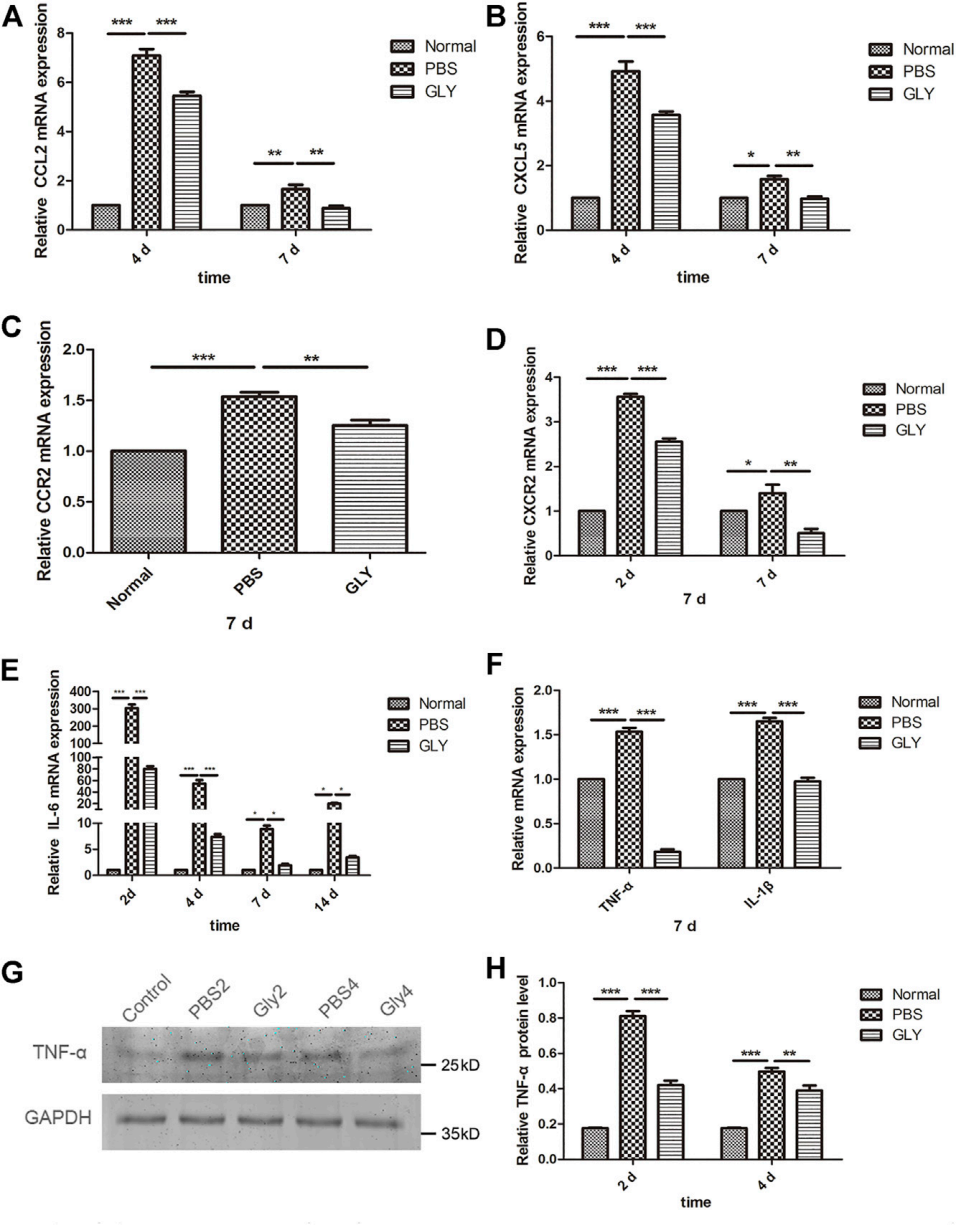
HMGB1 mediates the expression of the inflammatory cytokines and chemokines. **(A–F)** mRNA expression levels of CCL2, CXCL5, CCR2, CXCR2, IL-6, TNF-α and IL-1β at the time points indicated. The results are expressed as mean ± SD from three independent experiments and each experiment was performed in triplicate. Data were analyzed using one-way ANOVA followed by Bonferroni’s multiple comparison test (*n* = 6/group). **(G)** Western blot assay of expression of TNF-α in the corneas of mice from different groups, and the quantified data are shown in **(H)** Data were analyzed using one-way ANOVA followed by Bonferroni’s multiple comparison test (*n* = 3/group). The results are shown as the mean ± SD and are representative of three independent experiments. *p < 0.05, **p < 0.01, ***p < 0.001.

The differences in neutrophil infiltration might result in the differences in chemokine expression among the groups. CXCL5 is one of the important neutrophil chemoattractants and pro-angiogenic factors; while, CCL2 (monocyte chemoattractant protein-1, MCP-1) is a potent chemokine for monocytes. Therefore, we examined the expression of CXCL5 and CCL2 as well as their receptors CXCR2 and CCR2, respectively. RT-qPCR results showed that the expression of CXCL5 and CCL2 was markedly increased in alkali-burned corneas. This upregulation was reduced by GLY treatment (Figures 6A,B). The expression of CXCR2 and CCR2 was consistently downregulated in the GLY group compared with PBS group (Figures 6C,D). Inflammatory cytokines IL-6, TNF-α, and IL-1β was increased in PBS-treated group and GLY treatment rescued the expression (Figures 6E,F). The reduction in TNF-α levels was further supported by Western blot analysis (Figures 6G,H). Our results showed that neutrophil migration towards the inflammatory tissue is regulated by HMGB1, implying that HMGB1 might be a critical mediator of a neutrophil-mediated amplification loop.

### HMGB1 Induced Myofibroblast Differentiation and Corneal Opacity Through activating TGF-β1 Release

**FIGURE 7:**
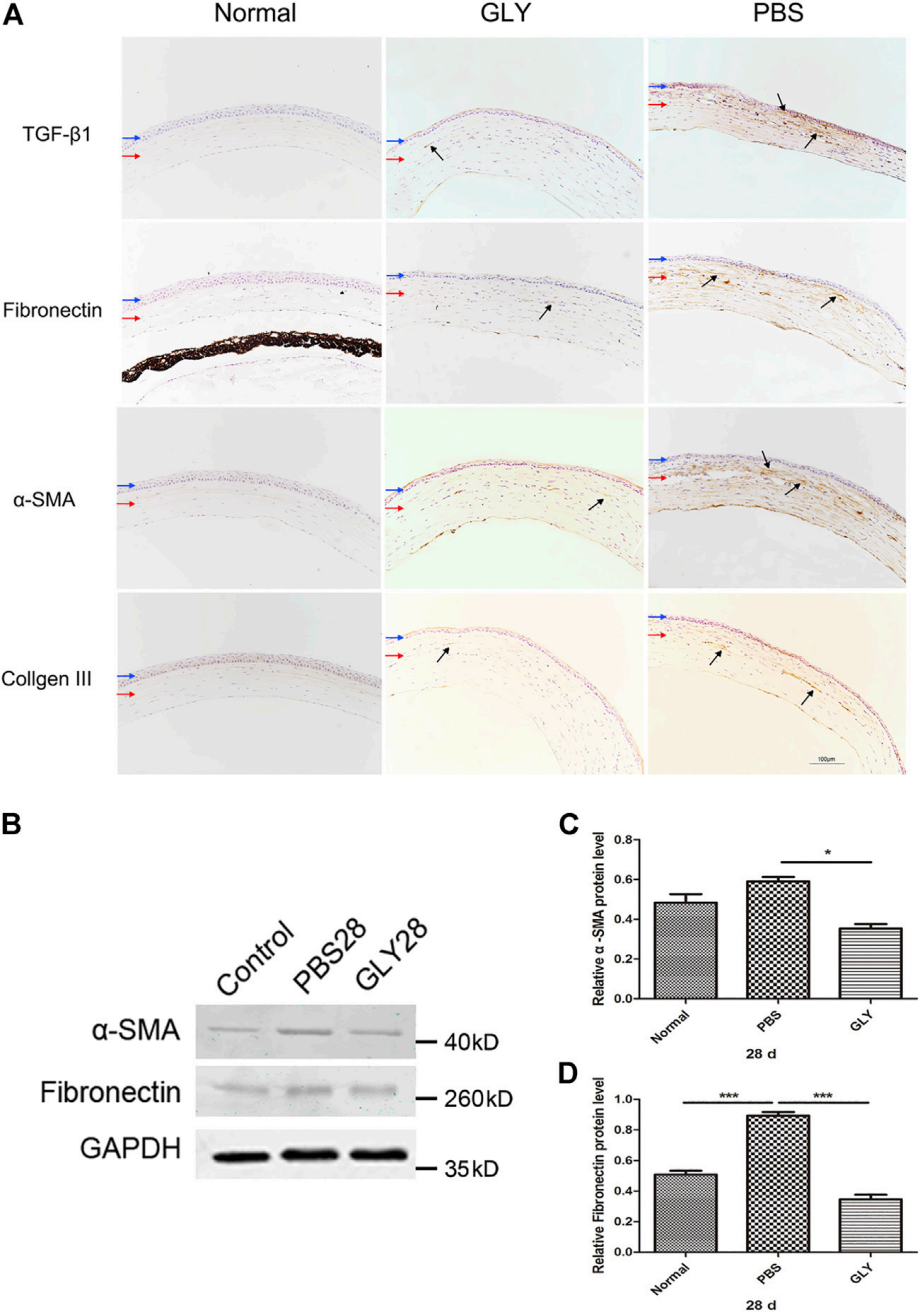
HMGB1 modulates myofibroblast differentiation and disorganized extracellular matrix generation. **(A)** The expression of TGF-β1, fibronectin, αSMA, and Collage III was examined by immunohistochemical staining (indicated by dark arrows). Blue and red arrows show corneal epithelium and cornea stroma, respectively. Scale bar = 100 μm. **(B)** Western blotting analysis of α-SMA and Fibronectin expression in corneas, and the quantified data are shown in **(C–D)** Data were analyzed using one-way ANOVA followed by Bonferroni’s multiple comparison test (*n* = 3/group). The results are shown as the mean ± SD and are representative of three independent experiments. *p < 0.05, **p < 0.01, ***p < 0.001.

The role of HMGB1 in regulating myofibroblast differentiation have been revealed in the process of fibrosis in lung, renal and myocardium (Li et al., 2014; Fan et al., 2016) In our study, we noticed that GLY treatment inhibited HMGB1 functions and reduced cornea opacity. To investigate the related signaling pathways through which HMGB1 generates corneal opacity, we used immunohistochemical staining for TGF-β1, Fibronectin, α-SMA and Collagen III in corneal tissue at 28 days after alkali-burn. Results showed that expression of TGF-β1, Fibronectin, α-SMA and Collagen III were markedly increased in PBS-treated group in comparison with that of the healthy controls, while the increase of these markers was reversed by GLY treatment (Figure 7A). Further analysis by western blot for α-SMA and Fibronectin showed the consistent results with that from immunohistochemical staining (Figures 7B–D). It is known that TGF-β1 activates the process of differentiation from fibroblasts to mature α-smooth muscle actin + myofibroblast, as well as the production of disorganized extracellular matrix components including Fibronectin and Collagen III. These results suggest that HMGB1 promote myofibroblast differentiation by inducing TGF-β1 release and results in an amount of fibrotic extracellular matrix, which causes cornea opacity, while GLY treatment reduces the generation of myofibroblasts and stromal opacity.

### Glycyrrhizin Reduced Injury–Induced miR-21 Expression, by Which Inhibited Keratocytes Differentiation

**FIGURE 8:**
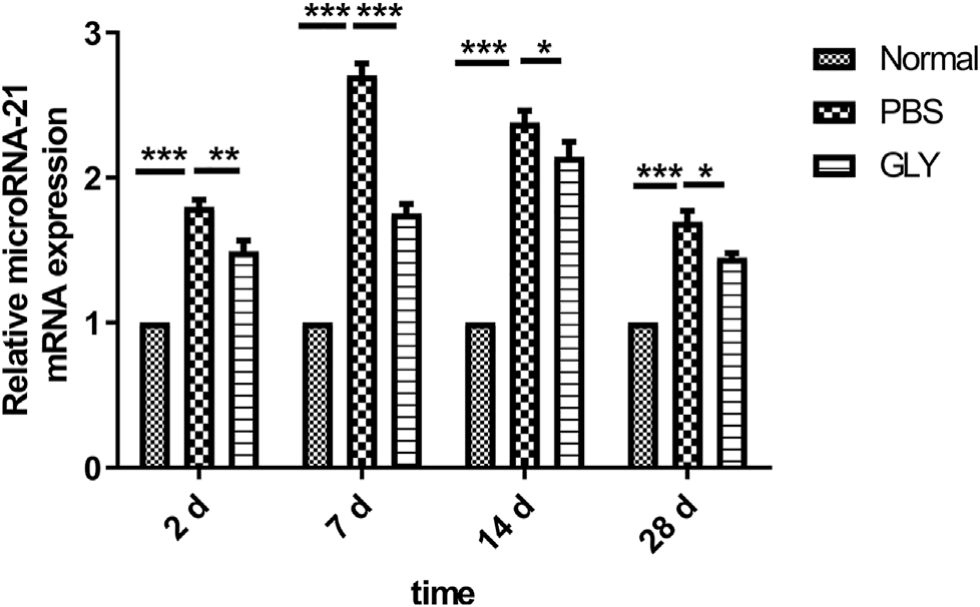
GLY attenuates alkali burn-induced upregulation of miR-21 in mouse cornea. At the indicated time points, miR-21 levels were measured in cornea samples by using qRT-PCR. Statistical analysis of data used a one-way ANOVA followed by bonferroni’s multiple comparison test and expressed as mean ± SD of six determinations (*n* = 6).

**FIGURE 9:**
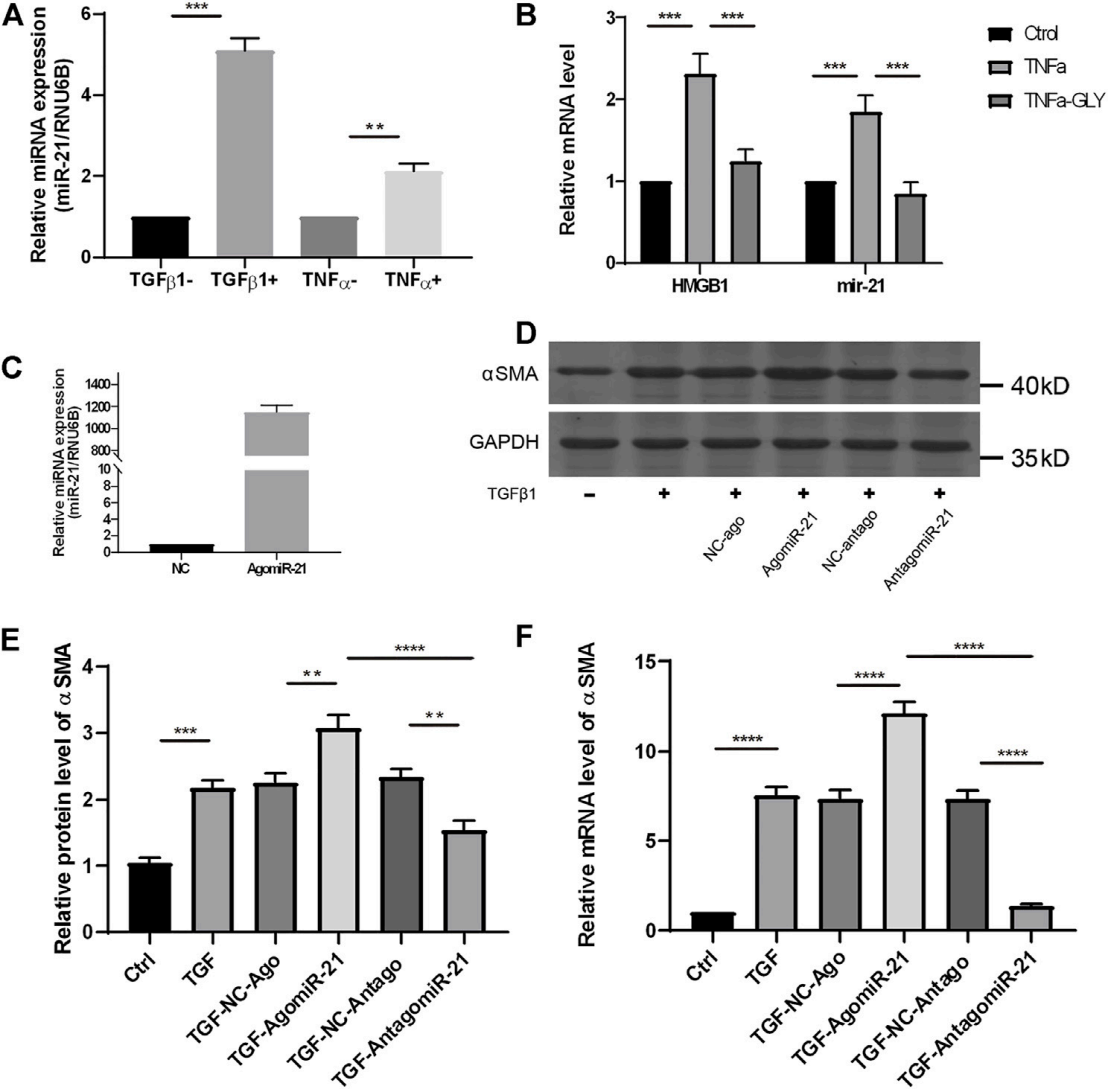
GLY reduces miR-21 expression in keratocytes, and blockade of miR-21 preventes keratocytes differentiation to myofibroblasts. **(A)** qRT-PCR shows significantly increased miR-21 level in HK stimulated by TNFα or TGF-β1. **(B)** mRNA expression levels of miR-21 and HMGB1 in HK treated by TNFα with or without GLY. **(C)** The expression level of miR-21 in HK after miR-21 agomir transfection. **(D–E)** Western blot and qRT-PCR were used to examine α-SMA expression after miR-21 transfection. HK were transfected with miR-21 agomir, antagomir, and their negative controls (NC) before treated with TGF-β1. Western blotting shows significantly increased of α-SMA in HK overexpressed miR-21 and the quantified data shown in **(E)** mRNA expression levels of α-SMA in HK **(F)** Data were analyzed using one-way ANOVA followed by Bonferroni’s multiple comparison test (*n* = 3/group). The results are expressed as the means ± SD of at least three independent experiments, and qRT-PCR was conducted in triplicate. *p < 0.05, **p < 0.01, ***p < 0.001.

We examined a possible mechanism by which GLY reduced stromal opacity in corneal injury model from the perspective of microRNA (miR). The expression of miR-21 was obviously increased in alkali burned corneas, while GLY significantly inhibited miR-21 expression (Figure 8). Therefore, we investigated the role of miR-21 in keratocytes wound healing *in vitro*. The expression of miR-21 was increased in either TNFα-induced inflammation or TGFβ1-induced fibrosis model *in vitro* (Figure 9A). GLY treatment significantly attenuated TNFα-induced upregulation of HMGB1 and miR-21 in keratocytes (Figure 9B). Next, we explored the effect of miR-21 on the transdifferentiation from keratocytes to myofibroblasts. We transfected keratocytes with miR-21 (AgomiR-21)/miR-21 inhibitor (AntagomiR-21) or its negative control (NC) and studied the effects of miR-21 on TGFβ1-induced myofibroblasts differentiation from keratocytes. Transfection of miR-21 substantially increased miR-21 expression (Figure 9C). As shown in Figures 9D–F, inhibition of miR-21 by transfected miR-21 antagomiR significantly attenuated TGFβ1-induced α-SMA expression in keratocytes at both the mRNA and protein level, while α-SMA expression was increased when miR-21 was overexpressed using the miR-21 agomir. Collectively, these findings indicate that HMGB1 inhibition can reduce miR-21 expression, which mediates keratocytes differentiation to myofibroblasts.

## Discussion

HMGB1 and the interplay with its ligands are illustrated in the process of several ocular diseases such as Aspergillus fumigatus keratitis (Jiang et al., 2019), acute glaucoma (Chi et al., 2015), chronic autoimmune uveitis (Yun et al., 2017), and diabetic retinopathy (Liu et al., 2019). GLY, one of the effective constituents of G. glabra, has been confirmed to perform various pharmacological actions, including anti-cancer, anti-inflammatory, anti-viral, and hepatoprotective effects. GLY intervenes interactions between HMGB1 and cell receptors through inactivating the downstream mitogen-activated protein kinase (MAPKs)/NF-κB signaling pathways, which result in reduced immune cell migration and cytokine release in damaged tissue (Tan et al., 2018). A recent study has shown that GLY is effective in inhibiting CNV supported by slit-lamp and H&E staining (Shah et al., 2018), whereas the molecular mechanism of GLY on CNV has not been understood. In consequence, we investigated whether GLY, a specific inhibitor of HMGB1, ameliorates the inflammatory corneal neovascularization by performing an alkali-burn mice model *in vivo*. Results showed that HMGB1 had a considerable function on corneal inflammatory neovascularization, and GLY significantly inhibited HMGB1 expression as well as the activation of NF-κB p65. GLY treatment effectively alleviated the inflammation, angiogenesis and corneal opacity in alkali-burned cornea. Furthermore, GLY significantly attenuated alkali burn-induced miR-21 expression, which modulated the transdifferentiation from keratocytes to myofibroblasts. Therefore, GLY treatment reduces corneal stroma fibrosis and improves corneal transparency.

Qin Lin et al. have elucidated the mechanism by HMGB1-TLR4 signal pathway regulating CNV (Lin et al., 2011). To date, the most receptors described for HMGB1 are ARGE, TLR4, TLR2, and CXCR4. Recent study reveals that GLY inhibits the activation of HMGB1/TLR4/NFκb signal pathway in radiation-induced acute lung injury (Zheng et al., 2020). Our results are consistent with theirs. Besides, we found that the mRNA level of TLR4 was increased in corneal injury including mechanical and chemical injuries, and glycyrrhizin reduced the TLR4 mRNA in cornea (data not shown). Therefore, it is necessary to investigate the effects of GLY on HMGB1 downstream pathway including RAGE and TLR4 by using the specific inhibitor and transgenic mice (RAGE^−/−^, TLR4-/4) in the future.

There is an interaction between inflammatory cells (specifically neutrophils and macrophages) and angiogenic growth factors (i.e., VEGF family) in inflammatory angiogenesis. Inflammation can trigger the development of CNV. HMGB1, as an extensively reported mediator, has been confirmed to aggravate inflammation through the regulation of proinflammatory cell infiltration as well as cytokine and chemokine release (Andersson et al., 2018). HMGB1 recruits monocyte through the RAGE/NF-κB signaling pathway, while inhibits monocyte apoptosis by TLR4/MAPK/extracellular signal-regulated kinase (ERK) signaling pathway (Tan et al., 2018). During the early inflammatory response, infiltrating neutrophils and monocytes produce a large number of inflammatory and pro-angiogenic cytokines, including VEGF. The factor VEGF has been identified as a major member of angiogenic regulators which is fetal for vessel formation and maturation of embryotic and adult tissues. Consistently, the present study demonstrated that HMGB1 triggered the release of inflammatory cytokines including IL-1, IL-6, TNFα, CCL2, and CXCL5, as well as the production of angiogenic factor VEGF. Moreover, application of GLY at a concentration of 2ug/ul can effectively reduce the inflammation and angiogenesis in the alkali-burned cornea.

Corneal under normal conditions is an avascular tissue, inflammation-induced hypoxia is more prominent in corneal tissue. There is also evidence that the inflammatory component independent of hypoxia may be important for HIF-1α activation. HIF-1α is known to bind to a hypoxia-responsive element (HRE) within the promoter of VEGF gene to upregulate VEGF expression. On the other hand, NF-κB is known to be the downstream targets of HIF-1α. By this positive feedback, the inflammatory and angiogenic response could be amplified. In the present study, we found that HIF-1α increased in alkali-burned cornea, accompanied by the activation of NF-κB, and upregulation of VEGF. GLY application significantly attenuated alkali burn-induced HIF-1α and NF-κB activation, as well as reduced the expression of VEGF and CD31, finally inhibited corneal neovascularization. These are consistent with a previous study where HMGB1 induced the expression of HIF-1α and angiogenesis in rheumatoid arthritis (Park et al., 2015).

CCL2 and CXCL5 are fundamental chemokines that participate in monocyte/neutrophil migration and infiltration in inflammation, while HMGB1 mediates to facilitate leukocytes and immune cells to liberate TNF-α to subsequently stimulate CCL2 and CXCL5 expression by vascular endothelium *in vitro* (Emilie, 2012). CCL2/CCR2 signaling is best known for its role in regulating macrophage recruitment and polarization during inflammation. CXCL5 bind CXCR2 to recruit neutrophils, to promote angiogenesis and remodel connective tissue. These factors are known angiogenic and inflammatory chemokines. It was recently reported that CCL2 and CXCL5, are family members of the most up-regulated genes in corneal neovascularization under the condition of inflammation (Mukwaya et al., 2016). These inflammatory chemokines amplify the cascade inducing chemotaxis of leukocytes and polarizing macrophages toward a pro-angiogenic phenotype. In the present study, GLY diminished CCL2 and CXCL5. Moreover, blockade HMGB1 by GLY, we also observed a reduction in the expression of CXCR2 and CCR2, which plays an important role in both angiogenesis and inflammation through neutrophil recruitment.

TGF-β1, a significant effect on the transformation of fibroblasts to myofibroblasts to generate fibrosis (Torricelli et al., 2016). In many fibrotic diseases, HMGB1 combines with such as RAGE, TLRs to activate NF-κB, mTOR and other pathways so as to regulate tissue regeneration, organ fibrosis, and inflammatory disorders (Lu et al., 2019). Furthermore, effective therapies to delay the development of fibrosis, such as anti-HMGB1 antibodies and gene therapies were accompanied by the reduction of TGF-β1 and HMGB1 infiltration. Consistent with these studies, our study showed that alkali-burn reactivated TGF-β1 generation and induced the development of mature myofibroblasts after injury. In addition, GLY decreased corneal haze opacity and disorganized extracellular matrix components (fibronectin and collagen III). Therefore, blockade of HMGB1 functions with GLY treatment contributes to the regulation of TGF-β1 expression and decreased myofibroblast, fibronectin, and collagen III generation.

Based on above work, we get further insights into the mechanism on the underling antifibrosis effect of GLY in keratocytes in the current study. We found that miR-21 might connect HMGB blockade to inhibit corneal fibrosis response. Similar to the condition observed in other tissue wound healing (Yan et al., 2020), the expression of miR-21 was increase in alkali-burned cornea. However, its expression was significantly reduced by GLY, the specific inhibitor of HMGB1 at both *in vitro* cellular level and the *in vivo* samples. This is consistent with the finding that HMGB1 induced miR- 21 expression in hepatocellular carcinoma (Chen et al., 2015). Moreover, we demonstrated that miR-21 plays a critical role in the anti-fibrosis effect of GLY on keratocytes differentiation to myofibroblasts. The finding in keratocytes is consistent with previous reports that miR-21 promotes cardiac fibroblast-to-myofibroblast transformation (Zhou et al., 2018).

The role of miR-21 in CNV has been reported (Yasar et al., 2020). In this study, they have demonstrated that VEGF expression is significantly increased in the corneal samples with CNV, compare with the controls. Meanwhile, statistically significant over-expression (Fold-regulation value >2) of miR-21, miR-126, and miR-150, as well as down-expression (Fold-regulation value <-2) of miR-181 were found in the same comparison. These results of VEGF and miR-21 are upregulated in CNV are consistent with ours. Furthermore, we have demonstrated the inhibitory effects of GLY on miR-21 expression and VEGF expression in alkali-burned cornea in the current study. As for the mechanism of miR-21 mediates VEGF expression and angiogenesis, recent studies have shown that miR-21 increases VEGF expression via ERK and AKT signal in vascular endothelial cells (Liu et al., 2011); miR-21 promotes angiogenesis by targeting SPRY2 and/or KRIT1 in osteoarthritis and colorectal cancer (Ma et al., 2020; He et al., 2021).

MiR-21 plays an important role in several pathophysiological processes related to wound healing, including inflammation and angiogenesis as well as fibrosis. Here the role of miR-21 may arise in the pathogenesis of corneal chemical injury and its relation to HMGB1. Our data verified the concordant reduction of HMGB1 and miR-21 by GLY treatment in the corneas after alkali burns. This is consistent with previous reports which showed that HMGB1 induced upregulation of miR-21 in hepatocellular carcinoma (Chen et al., 2015). Most of the available data suggest that miR-21 expression is maintained by transcriptional and post-transcriptional regulation. TGF-β1 has been reported to induce miR-21at the post-transcriptional level in a Smad-dependent manner (Davis et al., 2008). It is reported that STAT3, a transcription factor, can be activated by IL6 and directly activate the transcription of miR-21 (Löffler et al., 2007). In this current study, we found that GLY significantly reduced the expression of IL-6 and TGF-β1 accompanied by decreased expression of miR-21 in the damaged cornea. It remains to be further investigated the regulatory mechanism of GLY on miR-21 expression and how it affects cornea wound healing.

In conclusion, CNV is a sight-threatening condition usually associated with inflammation, hypoxia, or limbal stem cell deficiency. Inflammation is a key factor in the pathophysiology of CNV. We demonstrate that GLY, a natural inhibitor of HMGB1 may have therapeutic potential for the chemical burn-induced corneal neovascular response and inflammation. Moreover, GLY contributes to the inhibition of HMGB1/NFκb and miR-21 that are involved in inflammation, neovascularization, and fibrosis. The corneal protective effect of GLY deserves further investigation and has potential clinical translational value.

## Data Availability

The original contributions presented in the study are included in the article/Supplementary Material, further inquiries can be directed to the corresponding author.
